# Meetings of Societies

**Published:** 1893-12

**Authors:** 


### MEETINGS OF SOCIETIES.

### Bristol Medico-Chirurgical Society.

*Annual Meeting, October 11th, 1893.*

Dr. E. Markham Skerritt, President, in the Chair.

After a vote of thanks to the retiring President (Dr. Markham Skerritt) had been unanimously passed, Mr. J. Greig Smith took the Chair, and read his inaugural address (see page 217), for which a cordial vote of thanks was given him. The Honorary Secretary, Assistant Editor, and Honorary Librarian read their annual reports, which were adopted. The following officers were chosen for the ensuing year:—President-Elect; Dr. A. J. Harrison: Honorary Secretary; Mr. Munro Smith: Honorary Librarian; Mr. L. M. Griffiths: Members of Committee; Dr. Michell Clarke, Mr. J. Dacre, Mr. W. H. Harsant, Dr. Aust Lawrence, Mr. A. W. Prichard, Dr. J. E. Shaw.

Dr. Watson Williams exhibited a drawing of a case of epithelioma of the soft palate, and made some remarks on the difficulty of distinguishing malignant growths from syphilitic disease in this position.

---

*November 8th, 1893.*

Mr. J. Greig Smith, President, in the Chair.

Dr. Michell Clarke showed a child's brain with distension of the ventricles and a granular condition of the ependyma. There was no tubercular deposit.

Dr. Aust Lawrence showed a specimen of fibroid degeneration of the placenta.

Mr. Greig Smith exhibited a blocked and inflamed Fallopian tube with thickened walls.

Dr. J. E. Shaw reported two cases of pneumo-thorax arising from unusual causes. The first was that of a man who had signs of advanced phthisis with pneumo-thorax and emphysema of the upper part of the chest wall. After death it was found that the first rib was necrosed, and the rough end had torn the lung tissue. The second case, a girl, was admitted to the Bristol Royal Infirmary with diarrhœa, distended abdomen, and signs of pneumo-thorax. After death, the lung was found collapsed, and with septic abscesses in its substance. The lateral sinus on one side was full of grumous, putrid clot, and this was undoubtedly the cause of the pyæmia. There were no symptoms during life which pointed to disease of the ear.

Mr. A. W. Prichard and Mr. H. F. Mole described a case of lupus of the face in a middle-aged woman, which had recurred after removal by scraping and cutting. Lantern-slides of the growth were exhibited, and showed great epithelial proliferation, suggestive in places of malignancy.

Mr. C. A. Morton showed an epithelial implantation cyst removed from a woman's thigh, caused by the entrance of a thorn years before.

Dr. R. G. P. Lansdown showed a biliary calculus which had caused death by lodging in the ileo-cæcal valve. Mr. F. P. Lansdown and Mr. R. G. Fendick also showed similar intestinal calculi.

G. Munro Smith, *Hon. Sec.*

---

### Plymouth Medical Society.

*Annual Meeting, November 15th,* 1893.

Mr. T. Leah, President, in the chair.

The following office-bearers were elected for 1893-94:—President; Mr. Connell Whipple: Vice-Presidents; Messrs. E. M. Russell Rendle (Plymouth), Leah (Stonehouse), and Thom (Devonport): Honorary Treasurer; Mr. J. Elliot Square: Honorary Librarian; Mr. C. E. Russell Rendle: Honorary Secretary; Mr. Reginald H. Lucy.

The annual dinner was held at the Duke of Cornwall Hotel, twenty-seven members and two guests being present. The membership of the Society now numbers forty-seven.

Reginald H. Lucy, *Hon. Sec.*

